# Comparison and validation of the Japanese score and other scoring systems in patients with peptic ulcer bleeding: A retrospective study

**DOI:** 10.1097/MD.0000000000034986

**Published:** 2023-08-25

**Authors:** Seong Hoon Kim, Hee Seok Moon, Seong Woo Choi, Sun Hyung Kang, Jae Kyu Sung, Hyun Yong Jeong

**Affiliations:** a Division of Gastroenterology, Department of Internal Medicine, Daejeon Veteran Hospital, Daejeon, South Korea; b Division of Gastroenterology, Department of Internal Medicine, Chungnam National University School of Medicine, Daejeon, South Korea.

**Keywords:** endoscopic intervention, endoscopy, gastrointestinal bleeding, mortality, peptic ulcer bleeding, sensitivity, specificity

## Abstract

Acute upper gastrointestinal bleeding (UGIB) is one of the most urgent medical conditions, with peptic ulcer bleeding (PUB) accounting for most gastrointestinal bleeding cases. The Japanese scoring system was developed to predict the probability of intervention in patients with UGIB, and it is more effective than other scoring systems, according to several studies. This study aimed to verify whether the Japanese scoring system is better than other scoring systems in predicting the probability of intervention when limited to PUB in patients with UGIB. We enrolled patients who presented with symptoms of UGIB and were diagnosed with peptic ulcers using endoscopy. The performances of the scoring systems in predicting patient outcomes were validated and compared using the receiver-operating characteristic curve analysis. Additionally, we used the chi-square test, Fisher exact test, and the *t* test to analyze the association between the patients characteristics and clinical outcomes. Of the 1228 patients diagnosed with peptic ulcers, 90.6% underwent endoscopy. rebleeding occurred in 12.5% of the patients, and 2.5% of the patients died within 30 days. The Japanese score was the most effective in predicting the need for endoscopic intervention for PUB. Sex, systolic blood pressure, hematemesis, syncope, blood urea nitrogen level, and the American Society of Anesthesiologists score were predictive factors for the probability of endoscopic intervention in patients with PUB. The Japanese score is an effective predictor of the probability of endoscopic intervention in patients with PUB.

## 1. Introduction

Acute upper gastrointestinal bleeding (UGIB) is one of the most urgent conditions in the emergency department.^[1,2]^ The incidence of UGIB is reported to be 37 to 172 per 100,000 adults, and peptic ulcer bleeding (PUB) is the most common cause of UGIB, accounting for 28% to 59% of UGIB cases.^[3,4]^ The Japanese score introduced in 2016 was developed to predict whether patients with UGIB would require endoscopic intervention.^[5]^ It can be divided into 7 variables based on patient medical history (use of antiplatelets), symptoms (syncope and hematemesis), and measured laboratory data [systolic blood pressure (SBP), hemoglobin (Hb), blood urea nitrogen (BUN), and estimated glomerular filtration rate].^[5]^ The Japanese score has been evaluated as an effective scoring system in Japan; however, it has not been systematically verified for Koreans. Recently, a few studies have compared the Japanese score with other scoring systems and verified the effectiveness of the Japanese score in patients with UGIB. Choi et al^[6]^ performed a retrospective study that compared the Japanese score with other scoring systems in patients with non-variceal upper gastrointestinal bleeding (NVUGIB) and found that compared to other scoring systems, the Japanese score was more effective in predicting endoscopic intervention but showed no superiority in predicting 30-day mortality or rebleeding. Therefore, this study aimed to determine whether the Japanese score is superior to other validated systems [age, blood test, and comorbidities (ABC) score; mental status, American Society of Anesthesiologists (ASA) score, and pulse rate (MAP); and Glasgow–Blatchford score (GBS)] in predicting clinical outcomes among patients with NUVGIB due to PUB.

## 2. Materials and Methods

### 2.1. Study design and population

Among the patients who visited the emergency room of the Chungnam National University Hospital between May 2012 and March 2022, those who presented with melena, hematemesis, or suspected UGIB due to reduced Hb levels based on laboratory data were included in this study. The exclusion criteria were; Absence of UGIB (lower gastrointestinal bleeding or melena-like stools due to iron ingestion); Bleeding after endoscopic treatment, such as endoscopic submucosal dissection or endoscopic mucosal resection; Bleeding from varices; Unknown bleeding sources; and Absence of follow-up within 30 days.

We collected patients data from the Clinical Data Warehouse of the Chungnam National University Hospital, and the values of each of the 4 scoring systems (Japanese score, ABC score, MAP score, and GBS) were calculated and compared. Variables such as patient characteristics (age, sex, comorbidities, and use of antiplatelet agents), initial vital signs (SBP and heart rate), symptoms (hematemesis, melena, syncope, and mental changes), laboratory data (Hb, BUN, and estimated glomerular filtration rate), endoscopic findings, endoscopic intervention, 30-day mortality, and presence of rebleeding were collected.

Ethical approval was obtained for all protocols from the local institutional review board (IRB approval number: CNUH 2023-02-058). The requirement for informed consent from the patients was waived owing to the retrospective nature of this study.

### 2.2. Study outcomes

The primary outcome was to verify which of the 4 scoring systems was superior in predicting the need for endoscopic intervention. Secondary outcomes were the comparison of the scoring system in predicting 30-day mortality, rebleeding, and defining predictive factors for endoscopic intervention. Endoscopic intervention was limited to treatments using argon plasma coagulation (APC), hypertonic saline-epinephrine (HSE), coagrasper hemostatic forceps, or hemoclips, regardless of the successful cessation of bleeding. The 30-day mortality was defined as death from any cause, with or without UGIB. Rebleeding was defined as the need for re-endoscopy owing to unstable vital signs and reduced hemoglobin level of ≥ 2 g/dL for a day.

### 2.3. Statistical analyses

The receiver-operating characteristic curves of the 4 scoring systems were plotted, and area under the curve (AUC) values were used to compare the ability to predict the need for endoscopic intervention, 30-day death, and rebleeding. To determine the predictive factors of endoscopic interventions, statistical analyses, such as the *t* test, Fisher exact test, and chi-square test, were used. Differences were considered statistically significant at a *P* value < .05.

## 3. Results

### 3.1. Patient characteristics

**Figure 1.**
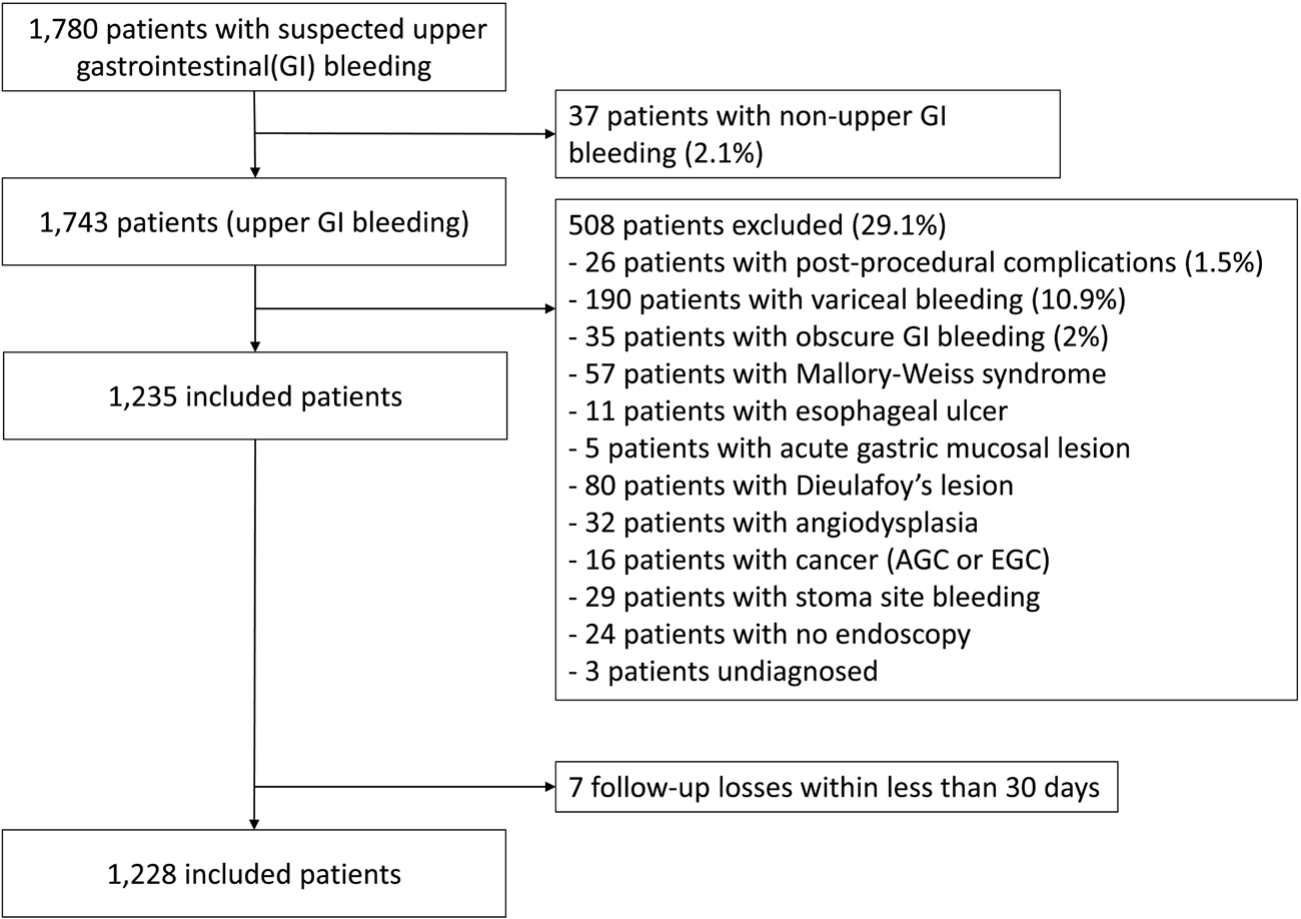
Flowchart of patient enrollment in this study. (AGC: advanced gastric cancer; EGC: early gastric cancer).

From May 2012 to March 2022, 1780 patients with suspected UGIB visited the emergency department of Chungnam National University Hospital. We excluded 515 patients from this study based on the exclusion criteria. Overall, 1228 patients were enrolled in this study (Fig. 1).

Of the 1228 patients, 929 (75.7%) were male, with an average age of 67 years. Among the patients who underwent endoscopy, gastric ulcer was the most common type of peptic ulcer (n = 828, [67.4%]), followed by duodenal ulcers (n = 387, [31.5%]) (Table 1). Overall, 1113 (90.6%) patients underwent endoscopy, while 30-day mortality and rebleeding occurred in 31 (2.5%) and 153 (12.5%) patients, respectively.

**Table 1:** Baseline characteristics of the study population (N = 1228).

Characteristic	Value
Age (yr; median [IQR])	67 (21)
Sex (male)	929 (75.7%)
Peptic ulcer	
Gastric ulcer	828 (67.4%)
Duodenal ulcer	387 (31.5%)
Combined	13 (1.1%)
Comorbidity	
Hypertension	618 (50.3%)
Diabetes	329 (26.8%)
Myocardial infarction	137 (11.2%)
Heart failure	76 (6.2%)
Cerebral infarction	197 (16.0%)
ICH	26 (2.1%)
Metastatic malignancy	77 (6.3%)
COPD	30 (2.4%)
ESRD	89 (7.2%)
Liver cirrhosis	66 (5.4%)
ASA score	
1	229 (18.6%)
2	358 (29.2%)
3	541 (44.1%)
4	100 (8.1%)
Mean (IQR)	2.4 (1)
Use of antiplatelets	460 (37.5%)
Use of anticoagulants	55 (4.5%)
Use of NSAIDs	63 (5.1%)
Initial vital signs (median [IQR])	
Systolic blood pressure (mm Hg)	116 (32)
Heart rate (beats/min)	92 (26)
Laboratory data (median [IQR])	
Hemoglobin level (g/dL)	9.1 (3.5)
Blood urea nitrogen level (mg/dL)	43.0 (26.0)
Albumin level (g/dL)	3.2 (.8)
Creatinine level (mg/dL)	1.20 (.18)
Scores (median [IQR])	
MAP	2.6 (2)
ABC	4.1 (4)
Japanese	2.4 (3)
GBS	10.1 (4)

ABC = age, blood tests and comorbidities, ASA = American Society of Anesthesiologists, COPD = chronic obstructive pulmonary disease, ESRD = end-stage renal disease, GBS = Glasgow–Blatchford score, ICH = intracranial hemorrhage, IQR = interquartile range, MAP = mental status-anesthesiologist score-pulse, NSAIDs = non-steroidal anti-inflammatory drugs.

Among 31 patients who died within 30 days, PUB accounted for the highest number of deaths with 13 cases, followed by 5 cases of both Liver Cirrhosis and Acute Respiratory Distress Syndrome, 3 cases of malignancy, 3 cases of Chronic Obstructive Pulmonary Disease, 1 case of heart failure (HF), and 1 case with an unknown cause of death.

### 3.2. Primary outcome

**Figure 2.**
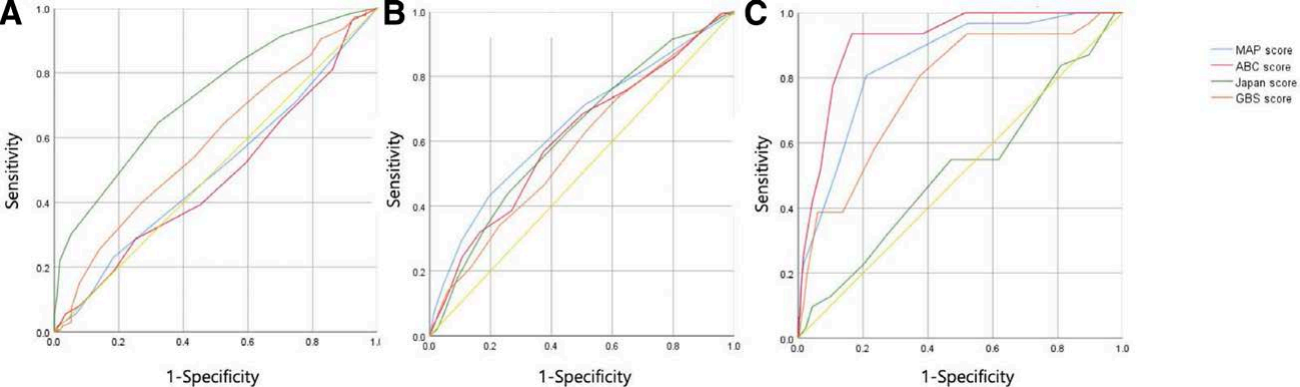
Receiver-operating characteristic curve of the Japanese score and other scoring systems. (A) Intervention, (B) Rebleeding, (C) 30-day mortality. ABC = age, blood test, and comorbidities, GBS = Glasgow–Blatchford score, MAP = mental status, American Society of Anesthesiologists score, and pulse rate, ROC = receiver-operating characteristic.

Figure 2 shows the receiver-operating characteristic curves of the 4 scoring systems and their ability to predict the need for endoscopic intervention, rebleeding, and 30-day mortality. The Japanese score was more effective than other scores in predicting the need for endoscopic intervention. The AUC value of the Japanese score was 0.724 (*P* < .001), and the values of other scoring systems were.478 (*P* = .43),.498 (*P* = .93), and.587 (*P* = .002) for the ABC score, MAP score, and GBS, respectively. The AUC values of the 4 scoring systems were not significantly different, which suggests that they do not differ in their ability to predict rebleeding. The AUC values were.625 (*P* < .001) for the Japanese score,.612 (*P* < .001) for the ABC score,.649 (*P* < .001) for the MAP score, and.583 (*P* < .001) for the GBS. The scoring systems, except the Japanese score, were all effective in predicting 30-day mortality, and the ABC score showed the best predictive ability among them. The AUC values were.914 (*P* < .001) for the ABC score,.847 (*P* < .001) for the MAP score,.769 (*P* < .001) for the GBS, and.517 (*P* = .74) for the Japanese score.

### 3.3. Secondary outcome

Predictive factors for the need for endoscopic intervention were male sex, age > 65 years, SBP < 100 mm Hg, hematemesis, syncope, BUN level ≥ 22.4 mg/dL, use of antiplatelet agents, and the ASA score in the univariate analysis. The multivariate analysis showed that male sex, SBP < 100 mm Hg, hematemesis, syncope, BUN level ≥ 22.4 mg/dL, and ASA score were related (Table 2).

**Table 2:** Factors predictive of endoscopic intervention based on univariate and multivariate analyses.

Variable	Category	Univariate analysis OR (95% CI)	*P* value	Multivariate analysis OR (95% CI)	*P* value
Sex	Male	1.836 (1.223–2.756)	.03	1.716 (1.113–2.647)	.02
Age (yr)	>65	1.619 (1.081–2.424)	.02		
Systolic blood pressure (mm Hg)	<100	3.025 (1.639–5.581)	<.001	2.511 (1.340–4.705)	.004
Diastolic blood pressure (mm Hg)	<60	1.584 (.992–2.529)	.05		
Heart rate (beats/min)	≥100	1.528 (0.990–2.359)	.06		
Hematemesis	Yes	4.256 (2.196–8.246)	<.001	4.478 (2.286–8.773)	<.001
Syncope	Yes	8.355 (1.151–60.650)	.04	9.945 (1.351–73.185)	.02
Altered mental status	Yes	9.525 (.829–30.011)	>.99		
Hemoglobin level (g/dL)	<10	1.022 (.682–1.533)	.92		
Blood urea nitrogen level (mg/dL)	≥22.4	2.349 (1.541–3.581)	<.001	2.622 (1.661–4.140)	<.001
Creatinine level (mg/dL)	>1.7	1.206 (.731–1.988)	.47		
Albumin level (g/dL)	<3.0	1.245 (.819–1.893)	.31		
INR	>1.5	1.078 (.508–2.287)	.85		
Diabetes	Yes	.900 (.588–1.378)	.63		
Hypertension	Yes	.821 (.558–1.207)	.32		
Myocardial infarction	Yes	.896 (.497–1.616)	.72		
Heart failure	Yes	1.219 (.518–2.872)	.65		
Cerebral infarction	Yes	.658 (.410–1.055)	.08		
ICH	Yes	.788 (.233–2.665)	.70		
Disseminated malignancy	Yes	.884 (.414–1.888)	.75		
COPD	Yes	1.458 (.343–6.201)	.61		
End-stage renal disease	Yes	1.227 (.553–2.722)	.62		
Liver cirrhosis	Yes	1.035 (.437–2.451)	.94		
Antiplatelet agents	Yes	.600 (.408–.883)	.009		
Anticoagulant agents	Yes	.837 (.350–1.998)	.69		
ASA score	≥3	.575 (.386–.858)	.007	.593 (.375–.939)	.03

ASA = American Society of Anesthesiologists, CI = confidence interval, COPD = chronic obstructive pulmonary disease, ICH = intracranial hemorrhage, INR = international normalized ratio, OR = odds ratio.

The predictive factors for 30-day mortality are presented in Table 3. In the univariate analysis, statistically significant factors associated with mortality were age > 65 years, syncope, altered mental status, Hb level < 10 g/dL, creatinine level > 1.7 mg/dL, albumin level < 3.0 g/dL, international normalized ratio > 1.5, HF, cerebral infarction, disseminated malignancy, chronic obstructive pulmonary disease, end-stage renal disease, Liver Cirrhosis, and ASA score. Multivariate analysis revealed predictors, such as albumin level < 3.0 g/dL, HF, and Chronic Obstructive Pulmonary Disease.

**Table 3:** Factors predictive of 30-day all-cause mortality based on univariate andmultivariate analyses.

Variable	Category	Univariate analysis OR (95% CI)	*P* value	Multivariate analysis OR (95% CI)	*P* value
Sex	Male	.668 (.311–1.436)	.30		
Age (yr)	>65	22.790 (1.193–6.524)	.02		
Systolic blood pressure (mm Hg)	<100	31.712 (.811–3.616)	.16		
Diastolic blood pressure (mm Hg)	<60	21.828 (.886–3.772)	.10		
Heart rate (beats/min)	≥100	1.833 (.897–3.746)	.10		
Hematemesis	Yes	.644 (.262–1.584)	.34		
Syncope	Yes	63.806 (1.513–9.577)	.005	8	<.001
Altered mental status	Yes	49.405 (1.741–50.803)	.009		
Hemoglobin level (g/dL)	<10	37.838 (1.861–33.009)	.005		
Blood urea nitrogen level (mg/dL)	≥22.4	1.520 (.526–4.388)	.44		
Creatinine level (mg/dL)	>1.7	.826 (.136–.599)	.001		
Albumin level (g/dL)	<3.0	230.349 (7.205–127.838)	<.001	815.241 (3.336–69.623)	<.001
INR	>1.5	44.668 (2.026–10.758)	<.001		
Diabetes	Yes	10.793 (.338–1.857)	.59		
Hypertension	Yes	10.707 (.343–1.455)	.35		
Myocardial infarction	Yes	1.953 (.787–4.848)	.15		
Heart failure	Yes	45.775 (2.491–13.389)	<.001	24.129 (1.547–11.015)	.005
Cerebral infarction	Yes	12.572 (1.192–5.549)	.02		
ICH	Yes	21.563 (.205–11.915)	.67		
Disseminated malignancy	Yes	43.806 (1.513–9.577)	.005	3	<.001
COPD	Yes	59.015 (3.200–25.401)	<.001	6.110 (1.635–22.843)	.007
End-stage renal disease	Yes	44.792 (2.078–11.051)	<.001		
Liver cirrhosis	Yes	35.626 (2.330–13.586)	<.001		
Antiplatelet agents	Yes	.916 (.435–1.930)	.82		
Anticoagulant agents	Yes	20.706 (.094–5.271)	.734		
ASA score	≥3	213.860 (3.293–58.347)	<.001	6	<.001

ASA = American Society of Anesthesiologists, CI = confidence interval, COPD = chronic obstructive pulmonary disease, ICH = intracranial hemorrhage, INR = international normalized ratio, OR = odds ratio.

The predictors of rebleeding are presented in Table 4. Univariate analysis verified that SBP < 100 mm Hg, diastolic blood pressure < 60 mm Hg, heart rate > 100 beats/minutes, hematemesis, Hb level < 10 g/dL, albumin level < 3.0 g/dL, international normalized ratio > 1.5, disseminated malignancy, end-stage renal disease, and ASA score were predictive factors. Multivariate analysis revealed that SBP < 100 mm Hg, heart rate > 100 beats/minutes, hematemesis, and albumin level < 3.0 g/dL were related to rebleeding.

**Table 4:** Factors predictive of rebleeding based on univariate and multivariate analyses.

Variable	Category	Univariate analysis OR (95% CI)	*P* value	Multivariate analysis OR (95% CI)	*P* value
Sex	Male	1.505 (.977–2.319)	.06		
Age (yr)	>65	1.269 (.898–1.794)	.18		
Systolic blood pressure (mm Hg)	<100	2.295 (1.611–3.269)	<.001	1.650 (1.077–2.528)	.02
Diastolic blood pressure (mm Hg)	<60	1.844 (1.300–2.616)	.001		
Heart rate (beats/min)	≥100	1.593 (1.130–2.247)	.008	1.545 (1.074–2.222)	.02
Hematemesis	Yes	1.977 (1.391–2.810)	<.001	1.856 (1.283–2.685)	.001
Syncope	Yes	1.184 (.611–2.296)	.617		
Altered mental status	Yes	2.573 (.698–9.489)	.16		
Hemoglobin level (g/dL)	<10	1.557 (1.063–2.281)	.02		
Blood urea nitrogen level (mg/dL)	≥22.4	1.163 (.738–1.834)	.52		
Creatinine level (mg/dL)	>1.7	1.218 (.783–1.894)	.38		
Albumin level (g/dL)	<3.0	2.410 (1.711–3.395)	<.001	1.631 (1.073–2.480)	.02
INR	>1.5	1.975 (1.155–3.378)	.01		
Diabetes	Yes	1.000 (.682–1.466)	>.99		
Hypertension	Yes	.861 (.613–1.209)	.39		
Myocardial infarction	Yes	1.071 (.632–1.815)	.80		
Heart failure	Yes	1.807 (.999–3.267)	.05		
Cerebral infarction	Yes	1.459 (.955–2.227)	.08		
ICH	Yes	.915 (.271–3.084)	.89		
Disseminated malignancy	Yes	2.115 (1.199–3.730)	.01		
COPD	Yes	1.787 (.719–4.445)	.21		
End-stage renal disease	Yes	1.944 (1.191–3.172)	.01		
Liver cirrhosis	Yes	1.437 (.735–2.810)	.29		
Antiplatelet agents	Yes	.928 (.653–1.321)	.68		
Anticoagulant agents	Yes	1.207 (.559–2.605)	.63		
ASA score	≥3	1.803 (1.267–2.565)	.001		

ASA = American Society of Anesthesiologists, CI = confidence interval, COPD = chronic obstructive pulmonary disease, ICH = intracranial hemorrhage, INR = international normalized ratio, OR = odds ratio.

Furthermore, we calculated the rebleeding rates based on different hemostatic methods, and the results are as follows: 25% for cases treated using HSE and hemoclips; 20.9%, APC and hemoclips; 18.9%, HSE and APC; 16%, hemoclips only; 13.5% APC only; 12.9% HSE only; 9.5%, HSE and hemostatic powder; 7.4%, hemostatic powder only; and 2.9%, combination of HSE, APC, and hemoclips.

## 4. Discussion

PUB is an emergency condition with different prognoses in patients depending on whether endoscopic intervention is performed.^[7,8]^ However, adequate endoscopic examination requires a sufficient fasting time. Moreover, when active bleeding continues, it is often difficult to evaluate the cause because of large clots in the gastric cavity.^[9–11]^ Therefore, the patient’s need for intervention, risk of rebleeding, and risk of death can be predicted before endoscopic examination, and the treatment efficiency can be improved regarding time and cost by reducing the need for intervention.^[9,12]^ Various scoring systems, such as the ABC score,^[13,14]^ MAP score,^[15]^ and GBS,^[16]^ have been developed to classify high-risk patients.^[17]^ Several studies have shown that the GBS is more effective than other scoring systems in predicting the need for endoscopic interventions.^[18]^ A multicenter study performed in several countries showed that the GBS outperformed the pre-endoscopic Rockall score and the AIMS65 score when applied to predicting the need for endoscopic interventions.^[18–21]^ Another study introduced the MAP (ASH) score as a new scoring system for predicting the need for intervention and mortality in patients with UGIB and reported that the GBS predicted the need for endoscopic intervention better than the AIMS65 score and pre-endoscopic Rockall score.^[15]^

However, according to recent studies, for patients aged > 65 years, the GBS and AIMS65 score are ineffective for predicting the need for endoscopic intervention in patients with UGIB. The GBS includes predicting the need for blood transfusion treatment in addition to predicting the need for intervention^[22,23]^; therefore, there is a limit to predicting the need for intervention alone. Furthermore, the GBS’ accuracy is poor in predicting endoscopic intervention in patients with low Hb levels.^,[5]^

Therefore, to replace the GBS, a new Japanese scoring system has been introduced in Japan that, unlike the GBS, does not include variables for predicting the need for blood transfusion treatment; thus, its predictability is considered superior. A study of patients with UGIB in Japan has proven the superiority of the Japanese scoring system.^[5]^ A previous study in South Korea demonstrated the superiority of the Japanese score in predicting the need for endoscopic intervention for patients with UGIB, except in patients with NVUGIB.^[6]^

In this study, when the patient group was limited to those with PUB, the Japanese score showed superior predictive ability for the need for endoscopic intervention (AUC value: .724, *P* < .001) when compared with other scoring systems, including the GBS (AUC value: .587, *P* = .002). Compared with that observed in Choi study, the AUC value of the Japanese score for predicting the need for endoscopic intervention in patients with PUB was higher than that in patients with NVUGIB (AUC value: .699, *P* < .001).^[6]^ Direct comparison of the 2 values is difficult, and whether the increased AUC value is statistically significant is unknown because the values were not calculated in the same patient group; however, the pathogenesis of peptic ulcers, among other variables contributing to the Japanese score, may be related to the overall increase in the AUC value of the Japanese score when limited to predicting the need for intervention for patients with peptic ulcers.

The scoring systems did not differ in predicting the probability of rebleeding; however, for 30-day mortality, the Japanese score showed less predictive ability than the other scoring systems. This finding is consistent with the results of recent studies.^[24,25]^

In addition, this study demonstrated that among the variables that comprise the Japanese score, the SBP, hematemesis, syncope, and BUN level are more relevant in predicting the need for endoscopic intervention than are other factors and that sex and ASA score are related factors in predicting the need for endoscopic intervention. Further multicenter research is needed in the future, as this study showed that the Japanese score may be supplemented.

Given that the rebleeding rate is significantly associated with mortality,^[2,26,27]^ it is crucial to analyze factors related to rebleeding. In this study, through multivariate analysis, we identified several factors, including SBP < 100 mm Hg, heart rate > 100 beats/minutes, hematemesis, and albumin level < 3.0 g/dL.

Furthermore, rebleeding rates varied depending on the hemostatic methods employed. The lowest rate was observed when a combination of HSE, APC, and hemoclips (2.9%) was used, whereas the highest rate was observed when a combination of HSE and hemoclips (25%) was used. While some studies have demonstrated the effectiveness of each hemostatic method,^[28,29]^ few studies have compared their effects on hemostasis.^[30–32]^ In this study, owing to uncontrolled variables and the varying choice of hemostatic method based on the severity and extent of bleeding, the direct comparison of rebleeding rates according to the hemostatic methods was challenging.

Considering the profound relationship between rebleeding and mortality, further studies may be necessary to analyze the hemostatic effects of the different hemostatic methods.

Our study had some limitations. First, it was a single-center retrospective study. Second, because it was performed at a higher-level general hospital with high-severity cases, endoscopy was performed in almost all patients presenting with signs of bleeding, indicating the possibility of selection bias. Finally, the endoscopist was not the same for every case, and the therapeutic intervention was determined by the discretion and clinical context of the attending endoscopist.

## 5. Conclusion

The AUC value of the Japanese score was higher than that of the ABC score, MAP score, and GBS in predicting the need for endoscopic intervention in patients with PUB. However, there were no differences among the scoring systems in predicting the probability of rebleeding, and the Japanese score was less predictive than the other scoring systems regarding the probability of 30-day mortality. Patient variables, including male sex, SBP < 100 mm Hg, hematemesis, syncope, BUN level > 22.4 mg/dL, and ASA score, were found to be predictors of the need for endoscopic intervention. Considering the limitations of this study, further multicenter prospective studies are necessary.

## Author contribution

**Conceptualization:** Hee Seok Moon.

**Data curation:** Seong Hoon Kim.

**Formal analysis:** Seong Hoon Kim.

**Investigation:** Seong Hoon Kim.

**Methodology:** Seong Woo Choi.

**Project administration:** Hyun Yong Jeong.

**Resources:** Sun Hyung Kang.

**Software:** Seong Woo Choi.

**Supervision:** Jae Kyu Sung.

**Validation:** Sun Hyung Kang.

**Visualization:** Seong Hoon Kim.

**Writing – original draft:** Seong Hoon Kim.

**Writing – review & editing:** Hee Seok Moon.
